# Supplementary material for: Integrating national open databases for a comprehensive view on food systems, environment sustainability and health in Brazil
Source: PLoS One. 2025 Nov 14;20(11):e0329353. doi: 10.1371/journal.pone.0329353 (PMC12617843; doi:10.1371/journal.pone.0329353)
Supplement: S1 File — Includes the datasets and metadata used in the PHFood Brazil integration process, structured in four phases: Phase 1 – Datasets of food production and land use from the Municipal Agricultural Production Information (PAM) by year; datasets from the National Water Agency (ANA); and the Brazilian Food Composition Table (IBGE). Phase 2 – Datasets from the Ministry of Agriculture and Livestock (MAPA), including the Phytosanitary Pesticides System (Agrofit). Phase 3 – Datasets from the National Plan to Control Residues and Contaminants (PNCRC/Vegetal) and the National Health Surveillance Agency (ANVISA). Phase 4 – Files from the Food and Nutrition Surveillance System (SISVAN) for adults, special conditions, elderlies, and teenagers, and the Family Budget Surveys (POF) for food acquisition data. (DOCX) [file pone.0329353.s001.docx]

Supplementary Material

**SM-Phase1**

Box 1 – Datasets of food production and land uses from the Municipal Agricultural Production Information (PAM) by years

| File Name | Access |
| --- | --- |
| PAM/tabela5457.xlsx | https://sidra.ibge.gov.br/pesquisa/pam/tabelas > Informações sobre culturas temporárias e permanentes > Numero 5457 > Unidade da Federação > Em Grande Região |

Box 2 – List of datasets from the National Water Agency – Brazil (ANA) by years

| File Names | Access |
| --- | --- |
| ANA_AtlasIRRIGACAO_sequeiro_municipio_2013-2017.xlsx, sheet = "2013ok"  ANA_AtlasIRRIGACAO_sequeiro_municipio_2013-2017.xlsx, sheet = "2014ok"  ANA_AtlasIRRIGACAO_sequeiro_municipio_2013-2017.xlsx, sheet = "2015ok"  ANA_AtlasIRRIGACAO_sequeiro_municipio_2013-2017.xlsx, sheet = "2016ok"  ANA_AtlasIRRIGACAO_sequeiro_municipio_2013-2017.xlsx, sheet = "2017ok" | https://metadados.snirh.gov.br/geonetwork/srv/eng/catalog.search#/metadata/15fca10a-d963-4f56-bb1a-1cdd21cef314 |

Box 3 – Food composition table from the Brazilian Institute of Geography and Statistics (IBGE)

| File Name | Access |
| --- | --- |
| liv50002_cd / tabelas / tabelacompleta.xls | https://biblioteca.ibge.gov.br/index.php/biblioteca-catalogo?view=detalhes&id=250002 > liv50002_cd > Tabelas > tabelacompleta |

**SM-Phase2**

Box 4 – Datasets of the Ministry of Agriculture and Livestock (MAPA) of Phytosanitary Pesticides System (Agrofit) with pesticides products information

| File Name | Access |
| --- | --- |
| agrofitprodutosformulado.xlsx  agrofitprodutostecnicos.xlsx  2a3f7e48-8b85-4757-b56c-05259cf27fa6.xlsx | https://dados.agricultura.gov.br/dataset/sistema-de-agrotoxicos-fitossanitarios-agrofit > Produto Formulado > Exportar  https://dados.agricultura.gov.br/dataset/sistema-de-agrotoxicos-fitossanitarios-agrofit > Produto Técnico > Exportar  https://mapa-indicadores.agricultura.gov.br/publico/extensions/AGROFIT/AGROFIT.html > Quantidade de Registros |

**SM-Phase3**

Box 5 – List of National Plan to Control Residues and Contaminants (PNCRC/Vegetal) datasets according to years of residues information

| File Names | Access |
| --- | --- |
| Resultados do PNCRC_Vegetal 2015_residuoAgro.csv  Resultados do PNCRC_Vegetal 2016_residuoAgro.csv  Resultados do PNCRC_Vegetal 2017_residuoAgro.csv  Resultados do PNCRC_Vegetal 2018_residuoAgro.csv  Resultados do PNCRC_Vegetal 2019_residuoAgro.csv  Resultados do PNCRC_Vegetal 2020_residuoAgro.csv | https://www.gov.br/agricultura/pt-br/assuntos/inspecao/produtos-vegetal/pncrcvegetal > Painel de resultados  > exportar |

Box 6 – Dataset of the National Health Surveillance Agency (ANVISA)

| File Name | Access |
| --- | --- |
| TA_MONOGRAFIA_AGROTOXICO.csv | https://www.gov.br/anvisa/pt-br/setorregulado/regularizacao/agrotoxicos/monografias > Painel de monográficas – Busca por culturas, classes agronômicas e ingredientes ativos > exportar |

**SM-Phase4**

Box 7 – Files from Food and Nutrition Surveillance System (SISVAN) with the files used for adults by years

| Files name | Access |
| --- | --- |
| CONS_FEIJAO.xlsx, sheet = 2015  CONS_FEIJAO.xlsx, sheet = 2016  CONS_FEIJAO.xlsx, sheet = 2017  CONS_FEIJAO.xlsx, sheet = 2018  CONS_FEIJAO.xlsx, sheet = 2019  CONS_FEIJAO.xlsx, sheet = 2020  CONS_FEIJAO.xlsx, sheet = 2021  CONS_FEIJAO.xlsx, sheet = 2022 | https://sisaps.saude.gov.br/sisvan/relatoriopublico/index > Arquivos Consolidados dos Acompanhamentos Registrados no Consumo Alimentar por Município > 2 ANOS OU MAIS > ADULTOS > Consumo de feijão 2015-2022 |
| CONS_FRUTA.xlsx, sheet = 2015  CONS_FRUTA.xlsx, sheet = 2016  CONS_FRUTA.xlsx, sheet = 2017  CONS_FRUTA.xlsx, sheet = 2018  CONS_FRUTA.xlsx, sheet = 2019  CONS_FRUTA.xlsx, sheet = 2020  CONS_FRUTA.xlsx, sheet = 2021  CONS_FRUTA.xlsx, sheet = 2022 | https://sisaps.saude.gov.br/sisvan/relatoriopublico/index > Arquivos Consolidados dos Acompanhamentos Registrados no Consumo Alimentar por Município > 2 ANOS OU MAIS > ADULTOS > Consumo de fruta 2015-2022 |
| CONS_VERD.xlsx, sheet = 2015  CONS_VERD.xlsx, sheet = 2016  CONS_VERD.xlsx, sheet = 2017  CONS_VERD.xlsx, sheet = 2018  CONS_VERD.xlsx, sheet = 2019  CONS_VERD.xlsx, sheet = 2020  CONS_VERD.xlsx, sheet = 2021  CONS_VERD.xlsx, sheet = 2022 | https://sisaps.saude.gov.br/sisvan/relatoriopublico/index > Arquivos Consolidados dos Acompanhamentos Registrados no Consumo Alimentar por Município > 2 ANOS OU MAIS > ADULTOS > Consumo de verduras e legumes 2015-2022 |

Box 8 – Files from Food and Nutrition Surveillance System (SISVAN) with the files used for special condition by years

| Files name (PREGNANT) | Access |
| --- | --- |
| CONS_FEIJAO.xlsx, sheet = 2015  CONS_FEIJAO.xlsx, sheet = 2016  CONS_FEIJAO.xlsx, sheet = 2017  CONS_FEIJAO.xlsx, sheet = 2018  CONS_FEIJAO.xlsx, sheet = 2019  CONS_FEIJAO.xlsx, sheet = 2020  CONS_FEIJAO.xlsx, sheet = 2021  CONS_FEIJAO.xlsx, sheet = 2022 | https://sisaps.saude.gov.br/sisvan/relatoriopublico/index > Arquivos Consolidados dos Acompanhamentos Registrados no Consumo Alimentar por Município > 2 ANOS OU MAIS > GESTANTES > Consumo de feijão 2015-2022 |
| CONS_FRUTA.xlsx, sheet = 2015  CONS_FRUTA.xlsx, sheet = 2016  CONS_FRUTA.xlsx, sheet = 2017  CONS_FRUTA.xlsx, sheet = 2018  CONS_FRUTA.xlsx, sheet = 2019  CONS_FRUTA.xlsx, sheet = 2020  CONS_FRUTA.xlsx, sheet = 2021  CONS_FRUTA.xlsx, sheet = 2022 | https://sisaps.saude.gov.br/sisvan/relatoriopublico/index > Arquivos Consolidados dos Acompanhamentos Registrados no Consumo Alimentar por Município > 2 ANOS OU MAIS > GESTANTES > Consumo de fruta 2015-2022 |
| CONS_VERD.xlsx, sheet = 2015  CONS_VERD.xlsx, sheet = 2016  CONS_VERD.xlsx, sheet = 2017  CONS_VERD.xlsx, sheet = 2018  CONS_VERD.xlsx, sheet = 2019  CONS_VERD.xlsx, sheet = 2020  CONS_VERD.xlsx, sheet = 2021  CONS_VERD.xlsx, sheet = 2022 | https://sisaps.saude.gov.br/sisvan/relatoriopublico/index > Arquivos Consolidados dos Acompanhamentos Registrados no Consumo Alimentar por Município > 2 ANOS OU MAIS > GESTANTES > Consumo de verduras e legumes 2015-2022 |

Box 9 – Files from Food and Nutrition Surveillance System (SISVAN) with the files used for elderlies by years

| Files name | Access |
| --- | --- |
| CONS_FEIJAO.xlsx, sheet = 2015  CONS_FEIJAO.xlsx, sheet = 2016  CONS_FEIJAO.xlsx, sheet = 2017  CONS_FEIJAO.xlsx, sheet = 2018  CONS_FEIJAO.xlsx, sheet = 2019  CONS_FEIJAO.xlsx, sheet = 2020  CONS_FEIJAO.xlsx, sheet = 2021  CONS_FEIJAO.xlsx, sheet = 2022 | https://sisaps.saude.gov.br/sisvan/relatoriopublico/index > Arquivos Consolidados dos Acompanhamentos Registrados no Consumo Alimentar por Município > 2 ANOS OU MAIS > IDOSOS > Consumo de feijão 2015-2022 |
| CONS_FRUTA.xlsx, sheet = 2015  CONS_FRUTA.xlsx, sheet = 2016  CONS_FRUTA.xlsx, sheet = 2017  CONS_FRUTA.xlsx, sheet = 2018  CONS_FRUTA.xlsx, sheet = 2019  CONS_FRUTA.xlsx, sheet = 2020  CONS_FRUTA.xlsx, sheet = 2021  CONS_FRUTA.xlsx, sheet = 2022 | https://sisaps.saude.gov.br/sisvan/relatoriopublico/index > Arquivos Consolidados dos Acompanhamentos Registrados no Consumo Alimentar por Município > 2 ANOS OU MAIS > IDOSOS > Consumo de fruta 2015-2022 |
| CONS_VERD.xlsx, sheet = 2015  CONS_VERD.xlsx, sheet = 2016  CONS_VERD.xlsx, sheet = 2017  CONS_VERD.xlsx, sheet = 2018  CONS_VERD.xlsx, sheet = 2019  CONS_VERD.xlsx, sheet = 2020  CONS_VERD.xlsx, sheet = 2021  CONS_VERD.xlsx, sheet = 2022 | https://sisaps.saude.gov.br/sisvan/relatoriopublico/index > Arquivos Consolidados dos Acompanhamentos Registrados no Consumo Alimentar por Município > 2 ANOS OU MAIS > IDOSOS > Consumo de verduras e legumes 2015-2022 |

Box 10 – Files from Food and Nutrition Surveillance System (SISVAN) with the files used for teenagers by years

| Files name | Access |
| --- | --- |
| CONS_FEIJAO.xlsx, sheet = 2015  CONS_FEIJAO.xlsx, sheet = 2016  CONS_FEIJAO.xlsx, sheet = 2017  CONS_FEIJAO.xlsx, sheet = 2018  CONS_FEIJAO.xlsx, sheet = 2019  CONS_FEIJAO.xlsx, sheet = 2020  CONS_FEIJAO.xlsx, sheet = 2021  CONS_FEIJAO.xlsx, sheet = 2022 | https://sisaps.saude.gov.br/sisvan/relatoriopublico/index > Arquivos Consolidados dos Acompanhamentos Registrados no Consumo Alimentar por Município > 2 ANOS OU MAIS > ADOLESCENTES  > Consumo de feijão 2015-2022 |
| CONS_FRUTA.xlsx, sheet = 2015  CONS_FRUTA.xlsx, sheet = 2016  CONS_FRUTA.xlsx, sheet = 2017  CONS_FRUTA.xlsx, sheet = 2018  CONS_FRUTA.xlsx, sheet = 2019  CONS_FRUTA.xlsx, sheet = 2020  CONS_FRUTA.xlsx, sheet = 2021  CONS_FRUTA.xlsx, sheet = 2022 | https://sisaps.saude.gov.br/sisvan/relatoriopublico/index > Arquivos Consolidados dos Acompanhamentos Registrados no Consumo Alimentar por Município > 2 ANOS OU MAIS > ADOLESCENTES  > Consumo de fruta 2015-2022 |
| CONS_VERD.xlsx, sheet = 2015  CONS_VERD.xlsx, sheet = 2016  CONS_VERD.xlsx, sheet = 2017  CONS_VERD.xlsx, sheet = 2018  CONS_VERD.xlsx, sheet = 2019  CONS_VERD.xlsx, sheet = 2020  CONS_VERD.xlsx, sheet = 2021  CONS_VERD.xlsx, sheet = 2022 | https://sisaps.saude.gov.br/sisvan/relatoriopublico/index > Arquivos Consolidados dos Acompanhamentos Registrados no Consumo Alimentar por Município > 2 ANOS OU MAIS > ADOLESCENTES  > Consumo de verduras e legumes 2015-2022 |

**SM-Phase4**

Box 11 – File from Family Budget Surveys (POF) used to access the food acquisition by population.

| File names | Access |
| --- | --- |
| Tabela2393.xlsx | https://sidra.ibge.gov.br/tabela/2393  select: variables, year, region, state, food |
